# Infrared Nanoimaging of Hydrogenated Perovskite Nickelate Memristive Devices

**DOI:** 10.1021/acsnano.3c09281

**Published:** 2024-01-10

**Authors:** Sampath Gamage, Sukriti Manna, Marc Zajac, Steven Hancock, Qi Wang, Sarabpreet Singh, Mahdi Ghafariasl, Kun Yao, Tom E. Tiwald, Tae Joon Park, David P. Landau, Haidan Wen, Subramanian K. R. S. Sankaranarayanan, Pierre Darancet, Shriram Ramanathan, Yohannes Abate

**Affiliations:** 1 Department of Physics and Astronomy, University of Georgia, Athens, Georgia 30602, United States; 2 Center for Nanoscale Materials, Argonne National Laboratory, Lemont, Illinois 60439, United States; 3 Department of Mechanical and Industrial Engineering, University of Illinois, Chicago, Illinois 60607, United States; 4 Advanced Photon Source, Argonne National Laboratory, Lemont, Illinois 60439, United States; 5 Center for Simulational Physics and Department of Physics and Astronomy, University of Georgia, Athens, Georgia 30602, United States; 6 School of Materials Engineering, Purdue University, West Lafayette, Indiana 47907, United States; 7 School of Electrical and Computer Engineering, University of Georgia, Athens, Georgia 30602, United States; 8 J.A. Woollam Co., Inc., Lincoln, Nebraska 68508, United States; 9 Materials Science Division, Argonne National Laboratory, Lemont, Illinois 60439, United States; 10 Northwestern Argonne Institute of Science and Engineering, Evanston, Illinois 60208, United States; 11 Department of Electrical & Computer Engineering, Rutgers, The State University of New Jersey, Piscataway, New Jersey 08854, United States

**Keywords:** neuromorphic devices, memristive devices, perovskite nickelates, phase change materials, near field microscopy, nano-FTIR

## Abstract

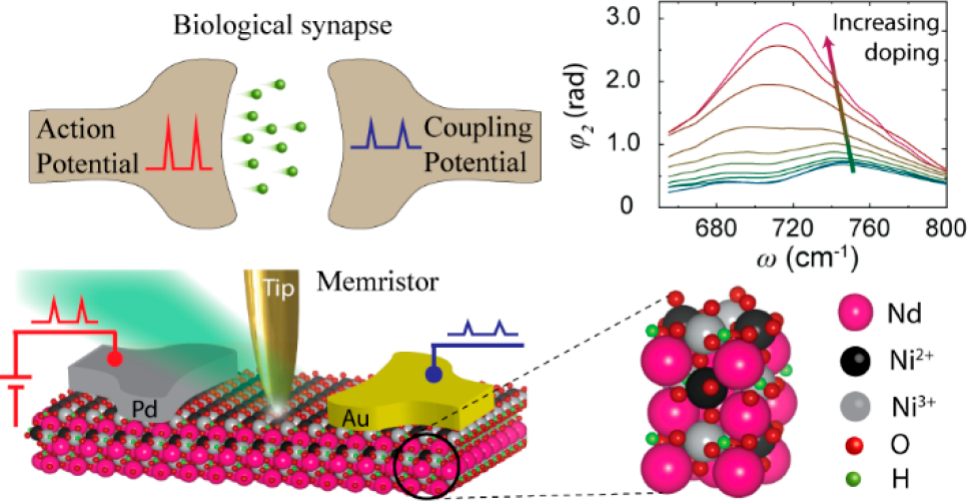

Solid-state devices made from correlated oxides, such as perovskite nickelates, are promising for neuromorphic computing by mimicking biological synaptic function. However, comprehending dopant action at the nanoscale poses a formidable challenge to understanding the elementary mechanisms involved. Here, we perform *operando* infrared nanoimaging of hydrogen-doped correlated perovskite, neodymium nickel oxide (H-NdNiO_3_, H-NNO), devices and reveal how an applied field perturbs dopant distribution at the nanoscale. This perturbation leads to stripe phases of varying conductivity perpendicular to the applied field, which define the macroscale electrical characteristics of the devices. Hyperspectral nano-FTIR imaging in conjunction with density functional theory calculations unveils a real-space map of multiple vibrational states of H-NNO associated with OH stretching modes and their dependence on the dopant concentration. Moreover, the localization of excess charges induces an out-of-plane lattice expansion in NNO which was confirmed by *in situ* X-ray diffraction and creates a strain that acts as a barrier against further diffusion. Our results and the techniques presented here hold great potential for the rapidly growing field of memristors and neuromorphic devices wherein nanoscale ion motion is fundamentally responsible for function.

Correlated oxides, specifically rare-earth nickelates (RNiO_3_ where R = rare-earth element), provide a promising platform to configure quantum phenomena at the atomic scale for neuromorphic devices and applications.^1−8^ In particular, the metal–insulator transition (MIT) can be modulated by interstitial doping to enable a reconfigurable synaptic unit.^8^ The hydrogen-doping-induced phase transition, coupled with the small size of protons, holds promise for achieving energy-efficient synaptic functions. Considerable research has been dedicated to understanding the intrinsic operation mechanisms involving defects in various memristive materials with the goal of improving device performance and facilitating the integration of devices into neuromorphic platforms.^2^ However, most studies to date have involved the motion of oxygen vacancies in oxides such as titania and hafnia where local dielectric breakdown is achieved under strong electric fields.^1,2,5^ On the contrary, in our system, we utilize an electronic phase transition induced by hydrogen donor doping in NdNiO_3_, proximal to a catalytic electrode. As the dopant front distributes under an electric field stimulus, the channel resistance changes due to migration of the phase transition boundary. In addition, while studies exist on electrical stimulus dependent measurement of global channel resistance,^2^ nanoscale imaging of the dielectric environment in an operating device has remained elusive. A detailed understanding of the mechanism of individual synaptic action and control of dopant related phenomena at the nanoscale remains a colossal challenge. This is because changes in charge concentration gradients occur within tiny length scales, and minute changes can produce immense effects in the transmitted signal. This presents outstanding challenges in both resolution and sensitivity to the localized charge and electronic structure. Such a measurement forms the principal contribution of this study coupled with first-principles theoretical treatment to better understand the experimental measurements.

In this work, we perform *operando* infrared nanoimaging of hydrogen-doped neodymium nickel oxide (H-NNO) devices to uncover how an applied electric field (E-field) can disrupt distribution, leading to localized nanoscale phases that govern the device’s overall electrical response. We map in real-space at the nanometer length scale the electric-field-driven dopant migration and electronic character of a H-NNO thin film. The hydrogen acts as a donor dopant by donating an electron to the Ni–O orbital manifold, and the proton resides as an interstitial species. The electron doping results in a massive metal to insulator transition. By controlling the concentration and distribution of the dopant, it is possible to obtain a multitude of resistance states for synaptic function.^9−11^ While multimodal characterization of H-doped nickelate films has been reported,^12−14^ the nanoscale perturbation of doped regions in an electric-field driven device remains an outstanding problem. Here, we found ordered steady states that exhibit alternating conducting and insulating stripe phases perpendicular to the field direction due to field-driven proton migration. Using density functional theory (DFT) calculations in conjunction with high-resolution X-ray diffraction (HRXRD) strain mapping and infrared spectroscopy of the vibrational properties, we reveal that this macroscopic state emerges because of the competition between the strain created by excess electron localization and the field-induced proton drift. By combining ellipsometry dielectric data and empirical calculations, we quantify the nanoscale modifications to the dielectric environment due to doping. We found that the migration of protons, driven by an applied field, caused a significant macroscopic increase in the material’s size, perpendicular to the direction of migration of the protons, by ∼6% due to the buildup of excess charge locally. At the same time, the E-field-driven migration also formed insulating barriers that run perpendicular to the flow of current.

## Results

**Figure 1:**
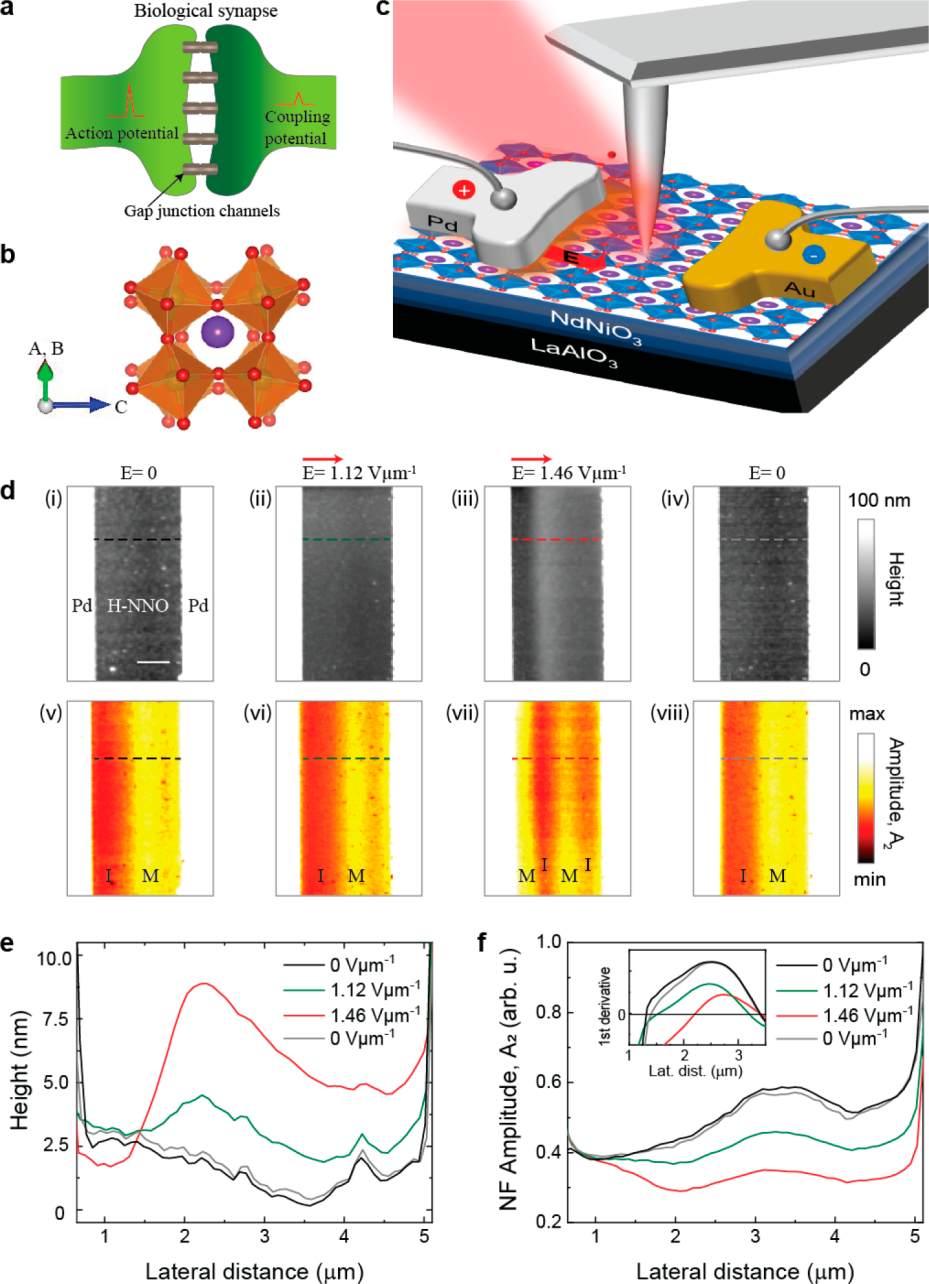
Schematics of the experimental setup, nanoimaging, and current–electric field relationships. (a) Schematic of a biological synapse that involves the transmission of electrical signals between two neighboring neurons. (b) Schematic of the crystal structure of H-doped NNO. The Nd atom is shown in purple at the center, O atoms are at the corners of octahedra, and Ni atoms are at the center of each octahedron. (c) Schematics of the s-SNOM experimental setup and a H-NNO/LAO synaptic device with Pd and Au electrodes. The metal coated probe tip with apex radius of ∼20 nm is illuminated by a mid-IR monochromatic laser for single frequency imaging or a broadband light source for nanospectroscopy. Back-scattered light from the tip–sample interface was detected and demodulated at higher harmonics of tip resonance frequency using interferometry methods as a function of bias voltage field. (d) Real-space nanoscale imaging of hydrogen migration in topography (i–iv) and s-SNOM amplitude (v–viii) images of doped H-NNO with Pd–Pd electrodes, obtained with illumination ν = 953 cm^–1^ as a function of applied E-field. Topography (d(i–iv)) and applied E-field dependent near-field amplitude images (b(v–viii)) for *E* = 0, 1.12, 1.46, and 0 V μm^–1^, respectively. Scale bar in d(i) is 2 μm. (e) Line profiles of topography and (f) amplitude along the lines marked on the topography and amplitude images shown in panel d. The inset in panel f shows first derivatives of the amplitude line profiles.

Figure 1a shows a schematic of a biological synapse and its gap junction which enables the exchange of currents.^15^Figure 1b shows schematics of the crystal structure of hydrogen-doped NdNiO_3_ (H-NNO). Pristine bulk NNO is a d-band electron-correlated pseudocubic perovskite structure with lattice constant *a* = 0.3807 nm. The R cations (Nd^3+^) are positioned in the cavities of ordered NiO_6_ octahedral networks. Figure 1c shows a schematic of a gated H-NNO sample that mimics a biological synapse (Figure 1a) and the scattering type scanning near-field optical microscopy (s-SNOM) setup used for nanoimaging and nano-FTIR (Fourier transform infrared) spectroscopy. The s-SNOM setup is based on a tapping mode atomic force microscope (AFM) where a metal coated cantilevered probe tip with an apex radius of ∼20 nm oscillates at a frequency of Ω ∼ 280 kHz with a tapping amplitude of ∼100 nm (see Methods for details).

Here, we demonstrate active manipulation of dopants using E-fields applied between electrodes in contact with the sample. Figure 1d shows a time series of topography and near-field optical amplitude images as a function of the applied external field. Since the NNO film is initially partially hydrogen doped, it exhibits coexistence of the insulating and metallic phases at zero bias, as shown in Figure 1d(v). The near-field amplitude images represent the value of the real part of the dielectric constant; hence the regions with a metallic phase (M) give higher signal (yellow) whereas the regions with an insulating phase (I) give lower signal (red). Note that the metallic phase (M) corresponds to low hydrogen doping while the insulating phase (I) refers to a high level of doping throughout this manuscript. The local phase modulation of H-NNO is the result of the inhomogeneous distribution of the dopant hydrogen atoms donating electrons to the Ni orbitals, modifying the electronic state of the d orbitals in NNO and generating a M/I boundary as reflected in the nanoscale s-SNOM amplitude contrast images (See also Supporting Information (SI) Figure S3).

During the field-dependent imaging experiments using a H-NNO device with Pd–Pd electrodes, the polarity and the magnitude of the E-field were kept constant for each image, and the recorded images are shown in Figure 1d. The action of the applied field between the Pd electrodes pointing from left to right creates two simultaneously measured changes in the sample: (i) the sample starts to physically expand as measured by AFM topography, and (ii) the dopants are pushed to form a new distribution between the electrodes as revealed by s-SNOM amplitude images. The topography images in Figure 1d and corresponding line profiles extracted from the topography images (see Figure 1e and SI Figure S1) reveal a dramatic structural expansion. We quantified this topographic change by measuring the height of the sample before and after E-field application and found up to a ∼6% expansion (see more details of topographic height measurement in SI Figure S1). When the E-field is turned off, the topography change is reversed and reverts to its original height demonstrating a piezo-like effect. Simultaneously, new M/I phases are created and captured by the s-SNOM amplitude images shown in Figure 1d. As the strength of the applied E-field is increased, alternating M and I phases appear clearly in the amplitude image (Figure 1d(vii)). The M or I phases form perpendicular to the current flow axis, presenting a transverse barrier to the movement of charge carriers that are driven by the electrical stimulus. This type of resistive switching is quite different from the more common conducting filament generation parallel to the current flow reported for other oxides exposed to E-fields.^16−18^ The amplitude image recovers its original M–I contrast when the applied field is turned off, as can be seen by comparing Figure 1d(v) and Figure 1d(viii). For the same device, we have also performed the topography and amplitude scans with decreasing applied field (SI Figure S8) and observed similar behavior.

The topographic height and the near field contrast changes at each pixel as a function of the applied field are also clearly captured by the line profile curves shown in Figure 1e,f, respectively. Comparison of these curves shows a one-to-one correlation of the change in topography to the amplitude contrast. Take, for example, the topography and the amplitude line profiles corresponding to *E* = 1.46 V μm^–1^ (red curves). At the highest topographic point at ∼2.2 μm lateral distance, we observe the lowest amplitude signal indicative of an insulating state that resulted due to proton accumulation. The correlation between the topographic height changes and the corresponding near-field contrasts of H-NNO are clearly shown in SI Figure S2. These changes induced by proton drift are plotted in reference to the initial state (i), shown in Figure 1d. At all electric field values (*E* = 1.12, 1.46, and 0 V/μm), changes in amplitude are consistently correlated with changes in height, indicating that the two mechanisms are concurrent in dopant drift. Pearson’s correlation coefficient, which measures the covariance of the two variables divided by the product of their standard deviations, has been used to quantify this correlation. Positive values in SI Figure S2 confirm this correlation between height and amplitude. Moreover, at increasing fields, we note the right drift of the stripes normal to the field, likely resulting from collective motion of hydrogen-rich regions. The inset in Figure 1f shows the first derivative of the amplitude line profiles. With increasing E-field, the location on the horizontal axis where the first derivative becomes zero (amplitude minima) is shifted to the right (away from the left electrode), indicating that the heavily doped stripe parallel to the electrode edge is moving to the right. Furthermore, the nanoscale variation of near-field amplitude has been analyzed and presented in SI Figure S3 by taking line profiles parallel to the AFM slow axis. By comparing three pairs of amplitude line profiles with separation of 80 nm (SI Figure S3b), we have shown that amplitude contrast change at such a small scale is clearly observable.

**Figure 2:**
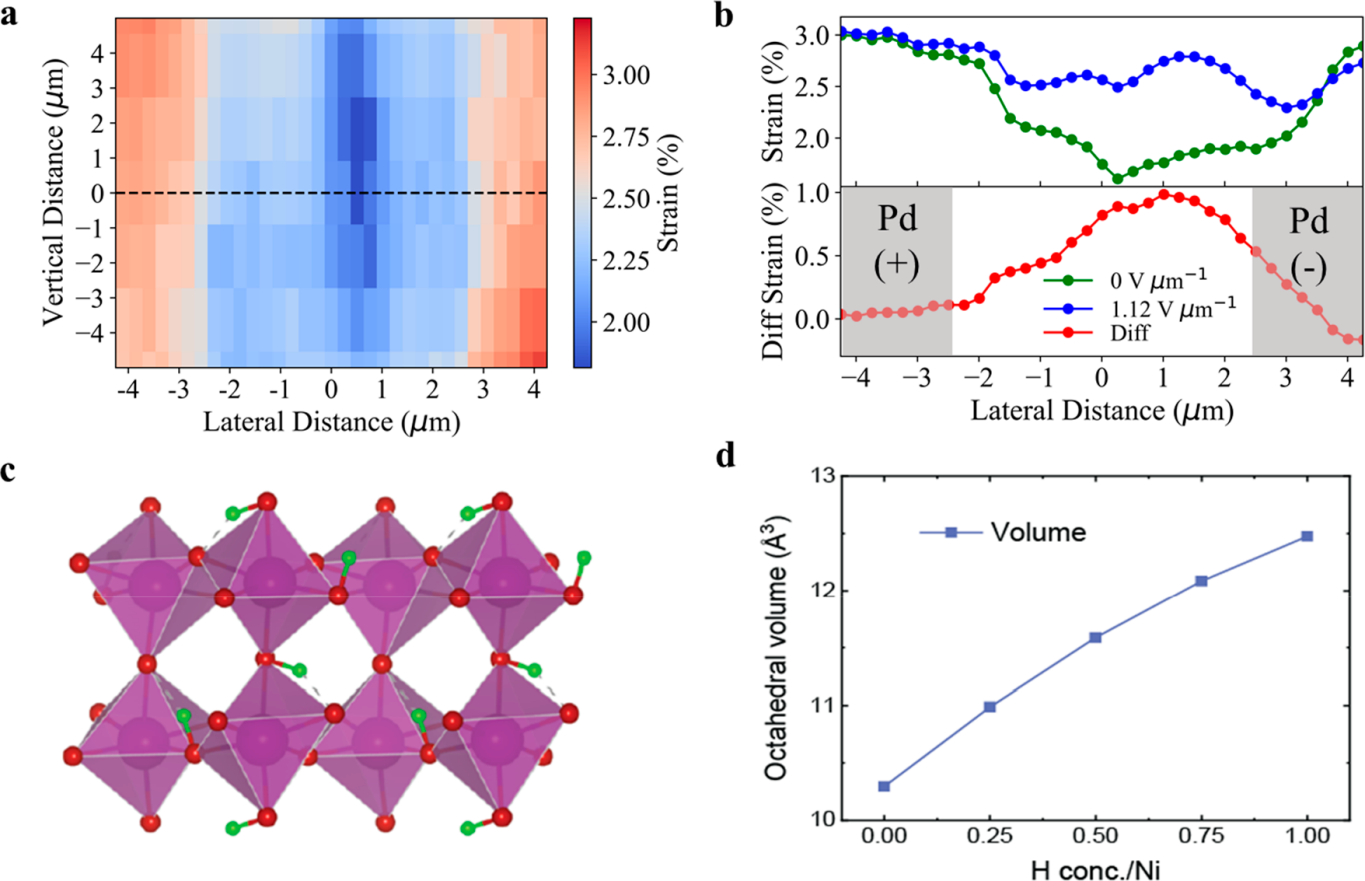
Structural changes of NNO due to H-doping and applied field measured by *in situ* X-ray diffraction (XRD) analysis of the electric-field-biased device with Pd and Pd electrodes. (a) Strain map of the device at 0 V μm^–1^ measured by the 004 reflection of the H-NNO film in the electrode-gap region, with respect to the out-of-plane lattice constant in the pristine film. (b) Change in the strain line profiles extracted along the black dashed line marked in panel a before and after applying 1.12 V μm^–1^. The vertical gray areas in panel b show the areas of electrodes. (c) Structure of H-NNO with one hydrogen per nickel. (d) Average octahedral volume as a function of hydrogen concentration.

To further explore how the structure of the NNO layer in these devices changes after H-doping, X-ray nanodiffraction imaging experiments were performed at the 7-ID-C beamline of the Advanced Photon Source (APS). The strain map across the gap (Figure 2a) was obtained by evaluating the measured lattice constant with respect to the lattice constant of the film outside the device far from the electrodes, which is close to that of the pristine NNO film.^12^ The positive strain implies that the H-doping of the sample leads to an out-of-plane expansion of the NNO, a behavior that has also been seen in other doped materials.^19^ This out-of-plane expansion should make the bonds weaker and softer, which is consistent with what was suggested by the s-SNOM measurements shown in Figure 1d. The s-SNOM measurements also showed that the higher the local H concentration is within the NNO film, the more the NNO film expands in the out-of-plane direction. Based on this, larger strain values under the electrodes (red regions in Figure 2a) imply that there is a higher H concentration under the electrodes compared to the H concentration in the gap of the device.

*In situ* electrical biasing experiments were also performed to understand how the H-NNO crystal structure changes with the DC bias. A series of diffraction images were collected along the black dashed line in Figure 2a to extract the strain profile across the gap before applying an E-field. A DC field of 1.12 V μm^–1^ was then applied across the device gap, and after waiting for the current through the device to stabilize, the X-ray diffraction measurements were repeated across the same region in the gap. The differences in the strain line outs are plotted in Figure 2b. The strain inside the gap increases as a result of an increase in the out-of-plane lattice constant. This lattice expansion is consistent with a field-driven hydrogen transport process, although the magnitude of change in strain is smaller than what is observed in ionic liquid gating experiments.^12^ Our observation unambiguously shows that lattice expansion correlates with the M/I phase changes revealed by s-SNOM when an E-field is applied (Figure 1d,e).

These observations are further supported by first-principles calculations. We investigated the effect of the hydrogen concentration on the volume expansion of H_*x*_-NNO with *x* values of 0, 0.25, 0.50, 0.75, and 1.00 per Ni atom using Density Functional Theory (DFT) calculations. Figure 2d reveals a nearly linear relationship between the H dopant concentration in NNO and the expansion of NiO_6_ octahedra, with changes of approximately 6.71%, 12.6%, 17.3%, and 21.2% for H per Ni atom ratios of 0.25, 0.5, 0.75, and 1.0, respectively. A corresponding optimized structure at one H per nickel is presented in Figure 2c. Upon H addition, we found that the electron from the hydrogen is transferred to a neighboring nickel atom. This results in a strong volume expansion of the nickel–oxygen octahedra as the hydrogen concentration increases. This excess charge localization process has been previously reported in related systems^20^ and has been associated with a significant renormalization of the electronic structure, with H-NNO becoming a wide band gap insulator. Importantly, the charge localization results in a significant volume increase driven by the octahedral expansion, as shown in Figure 2c. Interestingly, we find that this volume expansion occurs independently of the metallic or insulating nature of the ground state in our simulations, suggesting that electron localization and the associated volume expansion will occur in all of the hydrogen-rich regions of the device.

**Figure 3:**
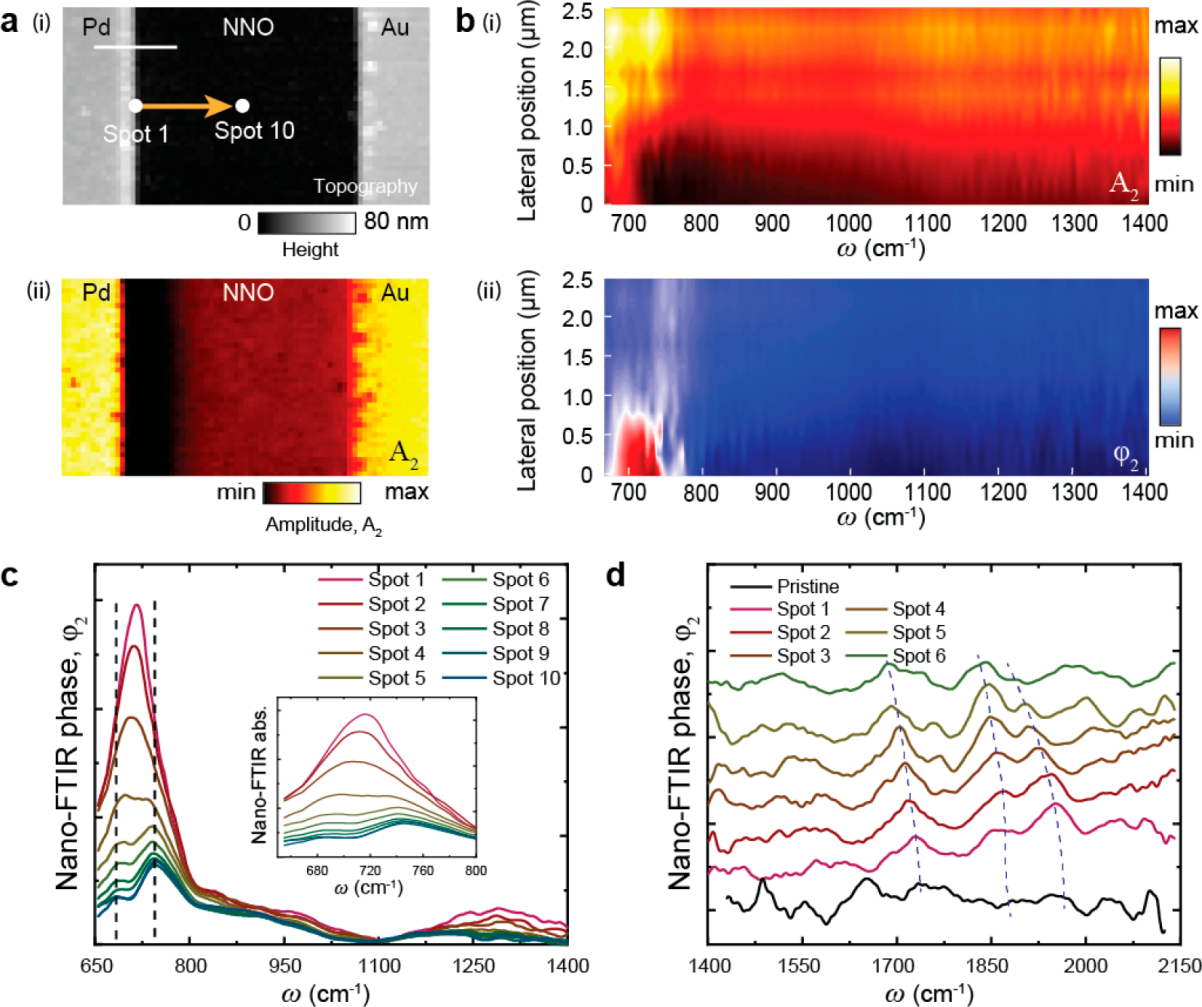
Nanospectroscopy of H-NNO revealing spectral evolution of vibrational modes as a function of doping level. (a) (i) Topography and (ii) near-field amplitude images of the H-NNO region between the Pd and Au electrodes, captured at illumination ω = 953 cm^–1^. (b) Hyperspectral (i) amplitude and (ii) phase map obtained along the 2 μm length marked by the yellow arrow in the topography image in panel a. (c) Extracted phase spectra from the hyperspectral phase map in panel b(ii) ranging from the most doped (spot 1) to least doped (spot 10) regions. (d) Phase spectra extracted from spot 1 to 6 together with a spectrum from a pristine NNO (black line). All spectra are normalized to that obtained on a Au reference surface and are vertically offset for clarity. Scale bar in panel a(i) is 2 μm.

Beyond the structural and nanoscale conductivity changes described above, the effect of hydrogen dopants can, in principle, be probed by the local spectroscopic signatures associated with the dopant vibrational states. However, thus far, the real-space spectroscopic signature of these states has remained elusive. We use hyperspectral nano-FTIR imaging to map the vibrational states of H-NNO and the dependence of these states on the dopant concentration. We employed a H-NNO sample with asymmetric electrodes (Pd–Au) since this configuration enables us to create an asymmetric dopant concentration gradient across the region between the electrodes, with the highest concentration of dopant closest to the Pd electrode (for catalytic hydrogen dissociation) and decreasing away from it toward the Au electrode. This gradient is clearly visible in the near-field amplitude contrast image (Figure 3a(ii)) and the 2D hyperspectral amplitude (Figure 3b(i)) and phase maps (Figure 3b(ii)).

The 2D hyperspectral phase and amplitude maps of the H-NNO sample were obtained by placing the tip of the microscope at 10 different equally spaced points along the yellow arrow line shown in the topography image in Figure 3a(i). The images show strong intensity and spatial variation, particularly in the spectral range of 650–780 cm^–1^. We extracted the phase spectral line profiles of each of the 10 points in the range of 650–1400 cm^–1^ and plotted them in Figure 3c. We also show phase spectra of just 6 points in the range 1400–2150 cm^–1^ in Figure 3d, as the spectra for points 7–10 were found to be too noisy in this frequency range. The spectral range of 640–780 cm^–1^ shows a clear dependence of intensity and peak position on location, which represents the dopant concentration. The phase peak at point 1, closest to the Pd electrode, shows the strongest peak intensity, and the signal intensity progressively decreases for points away from the Pd electrode.

The vibrational spectra of H-NNO shown in the spectral range of 600–2150 cm^–1^ are complicated by the presence of overlapping metal–oxygen and metastable vibrational modes that arise due to the hydrogen doping. To further understand the nature of these modes, we computed the phonon density of states using density functional perturbation theory calculations (DFPT) of H-NNO. As shown in Figure 2c, we consider the orthorhombic phase (*Pbnm*) of NNO with a concentration of hydrogen varying from 0.25 to 1 hydrogen per nickel in a 16 nickel atom periodic approximant. Complete computational details are given in the Methods section.

**Figure 4:**
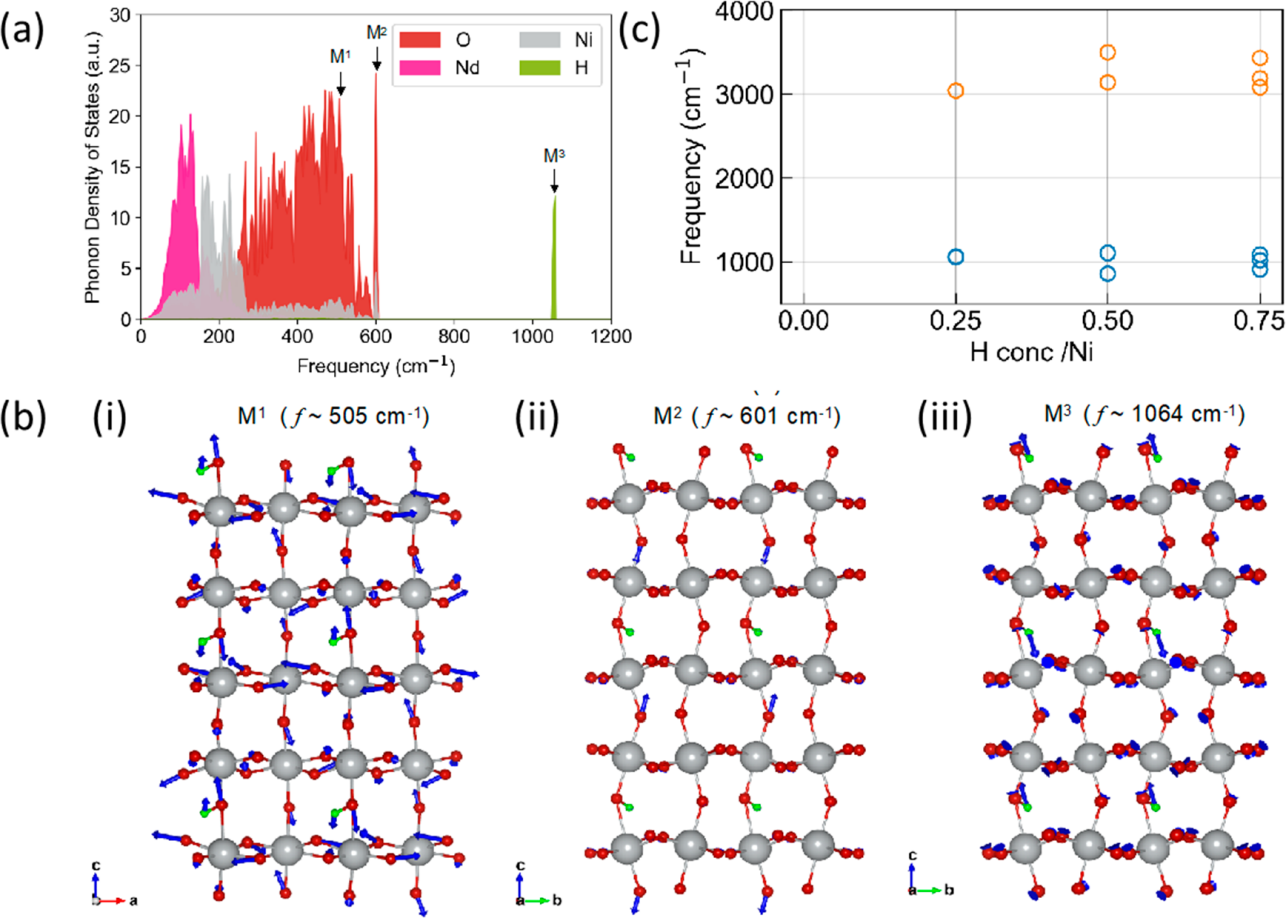
DFT analysis of H-doping effects on the vibrational modes of NNO. (a) Phonon density of states for 0.25H-NNO. (b) Phonon eigenvectors (depicted by blue arrows) illustrating modes with frequencies of (i) 505 cm^–1^, associated with the collective motion of NiO_6_ octahedra, (ii) 601 cm^–1^, indicating the vibration of O atoms, and (iii) 1064 cm^–1^, reflecting the vibration of H atoms. Additionally, panel c demonstrates the observed vibrational hybridization and spectral broadening upon the addition of H.

Computed zone-centered phonon frequencies and the corresponding decomposition of the vibrational eigenstates are given in Figure 4a for one representative hydrogen concentration of 0.25H/Ni (the phonon density of states for hydrogen-free NNO and other hydrogen concentrations is shown in SI Figure S6). Our calculations predict a nearly continuous phonon spectrum up to ∼600 cm^–1^. This prediction agrees with the results of the experimental measurement shown in Figure 3c. The heavier vibrational modes associated with the Nd- and Ni-dominated optical branches have frequencies up to 200 and 300 cm^–1^, respectively, while the 300–600 cm^–1^ frequency range is dominated by collective motions of the oxygen octahedra (e.g., mode M^1^ in Figure 4a). The M–O, M–M–O, and M–O–M (M = Ni, Nd) stretching vibration modes are also expected to be present in the experimental broad absorption band near 600 cm^–1^ (Figure 3e).

The addition of hydrogen results in two major changes in the vibration spectrum. First, OH stretching modes appear at higher frequencies, with strong variability of the frequency with respect to the hydrogen configuration and concentration, as shown in SI Figure S6b (3000 cm^–1^ for the longitudinal and ∼ 1064 cm^–1^ for the transverse motions for the configuration in Figure 4b). Second, when placed near an oxygen, the hydrogen perturbs the octahedral motion, resulting in high-energy modes of the unbound oxygens (see mode M^2^ in Figure 4b) slightly above the oxygen vibrational continuum. The maximum frequency observed in pristine NNO is 596 cm^–1^ (shown in SI Figure S6), while an oxygen dominated mode appears at 601 cm^–1^ in the H_0.25_NdNiO_3_ system. While the frequencies associated with hydrogen motion are likely to strongly vary in this system with many metastable states,^21^ our calculations clearly indicate that vibrational modes with frequencies in excess of 700 cm^–1^ are associated with OH stretching modes.^22−25^

These calculations agree with experimental observations of increased intensities of the broad peaks in the frequency range up to around 800 cm^–1^ with H dopant concentration. The ionic attachment of hydrogens to oxygen means that several combinations of O–H stretch and intercalated hydroxyl groups can be generated in the range 1400–2150 cm^–1^ as shown in Figure 3d. Even in the absence of explicit configurational disorder, we find that *x* > 0.25 concentrations lead to significant vibrational hybridization and spectral broadening. In particular, we find that hybridization of the OH modes of neighboring octahedra results in vibrational mode splitting on the scale of 100 to 350 cm^–1^. These modes appear to shift to lower energy with increasing dopant concentration. The spectral range 1600–1950 cm^–1^ is expected to be dominated by dopant related stretching and bending modes, and strikingly the peaks show a general trend of red shift as the concentration of the dopant increases (moving from Spot 10 to Spot 1). This is expected since strain increases with dopant concentration, which results in a spectral red shift and confirms dopant-based vibrations, as predicted by DFPT.

**Figure 5:**
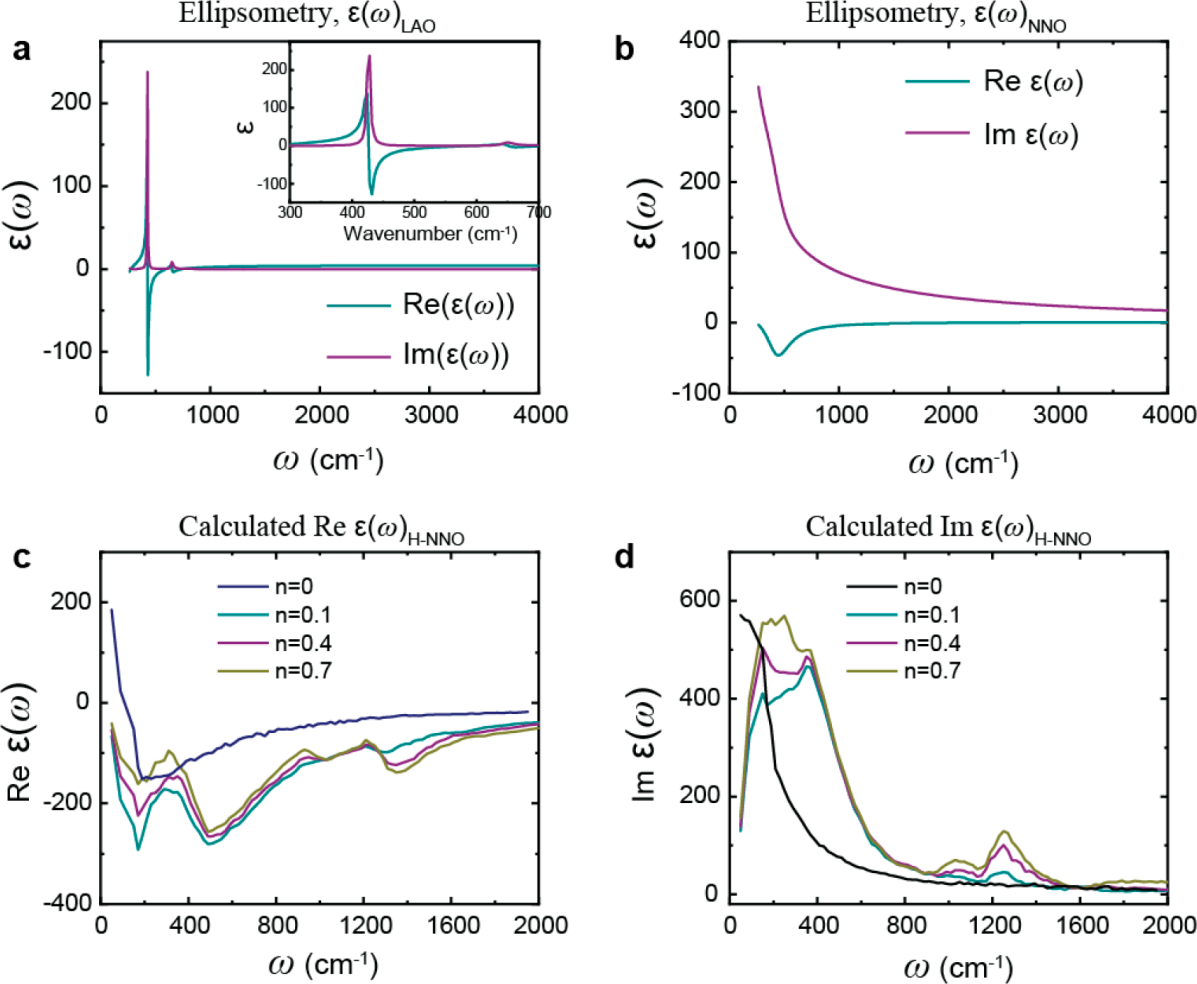
Experimental far-field ellipsometry real (green) and imaginary (purple) parts of the dielectric function of (a) LAO and (b) pristine NNO on the LAO substrate in the frequency range 400–4000 cm^–1^. Inset in panel a shows the zoomed in region from 300 to 700 cm^–1^. Theoretical calculations of (c) real and (d) imaginary dielectric functions of NNO at different doping levels, *n* = 0, 0.1, 0.4, and 0.7. The simulations were done at interaction parameters, ϵ_LJ_ = 1.54 × 10^–23^ and σ = 2 × 10^–10^ (see Methods section).

The response of H-NNO to the interaction with light is characterized by the complex-valued local dielectric function (ε(ω)) of the sample. The near-field vibrational absorption spectra (φ_*n*_) presented in Figure 3 can be directly assigned based on the imaginary part of the dielectric function (Im ε(ω)) of H-NNO. This is due to the direct proportionality of the near-field phase (φ_*n*_) to the Im ε(ω) for weak oscillatory modes, such as the dopant-induced vibrational modes that we analyze in this work. As such, mapping the dielectric function of H-NNO will provide direct information about the origin and assignment of the vibrational modes shown in Figure 3. To that end, we performed ellipsometry measurements in the frequency range from 400 to 4000 cm^–1^, using an IR-VASE ellipsometer (J. A. Woollam Co., Lincoln, NE) of pristine NNO on a LAO substrate and the pristine LAO substrate. Samples were mounted on a precision rotation stage, and a data set was acquired at a spectral resolution of 8 cm^–1^. After the measurement, the data was fit using standard numerical analysis methods (similar to Jellison^26,27^ and also Herzinger^28^) using the LAO optical function for the substrate. The real Re ε(ω) and imaginary Im ε(ω) parts of the dielectric function (ε(ω)) of NNO were calculated with a resistivity-scattering time Drude model^29^ plus a Lorentz oscillator to account for the additional absorption centered around 430 cm^–1^. The WVASE program from Woollam et al.^29^ was used to build the model and fit the data. Figure 5a,b shows the real (green solid line) and imaginary parts (purple solid line) of the dielectric functions extracted from ellipsometry for the LAO substrate and the NNO/LAO sample, respectively.

We note, however, that it is extremely challenging to perform ellipsometry measurements on NNO (H-NNO) with various dopant levels and to quantify the nanoscale changes of ε(ω) due to both sensitivity and resolution issues. To circumvent this difficulty, we implemented a simulation methodology that we recently developed to acquire information about the nanoscale ε(ω) of H-NNO.^30^ Using our model, we first reproduced the undoped dielectric function of NNO acquired from ellipsometry measurement (*n* = 0 lines in Figure 5c-d) and then predicted changes to its response at different dopant levels, *n* = 0.1, 0.4, and 0.7. This method considers harmonic interactions between bonded atoms and uses a combination of Langevin dynamics and Monte Carlo methods to investigate the frequency dependent dielectric function of a sample (see ref (30) for details). The undoped dielectric function of NNO was reproduced by fitting appropriate harmonic coupling constants, as well as damping parameters, to correspond to experimental results (Figure 3c,d). To model the effects of dopants in the system, we randomly placed particles interstitially throughout the lattice, where they interact with surrounding particles via a Lennard-Jones potential (see Methods section for details). The dopant level is controlled with a parameter *n* ∈ [0,1], which describes the number of dopants per unit cell in the system.

We vary the dopant concentration to explore broad feature modulations seen in the resultant calculated dielectric function. We found that increasing the dopant concentration produces a new peak in the dielectric function (Im ε(ω)) in Figure 5d) that increases in intensity as the dopant concentration increases, which is similar to what is observed in the experiment (Figure 3). When we allow the dopants in the system to couple to the lattice in a more complex way by including bond-angle and bond-length interactions with the surrounding crystal ions, we see multiple new peaks emerging. This indicates that the origin of the vibrational peaks is directly related to changes in Im ε(ω) due to both the dopant level (*n*) and the changes in the corresponding coupling strengths of the dopants. These 3-body potentials also correspond to the bond bending and stretching modes described above in the DFPT calculations (Figure 4), reinforcing this interpretation. We also leverage Monte Carlo techniques to investigate structural distortion induced by the inclusion of these dopants in our model. We allow the dopant particles to interact with the ions and allow the system to relax into its minimum free-energy configuration. Once we have established that the system has sufficiently relaxed, we calculate the total volume change of the system and perform such calculations over a variety of interaction parameters, as well as dopant levels, to ascertain overall trends. We find that the increase in dopant concentration leads to an overall increase of average unit cell volume, an effect which corresponds to the DFT calculation as well as the X-ray and s-SNOM measurements that we have described above in Figure 3. Furthermore, using the dielectric values obtained from the above-mentioned calculations, we generated the near-field amplitude and phase spectra using an extended finite-dipole model^31^ for NNO samples with different doping levels, and the results are presented in SI Figure S5.

**Figure 6:**
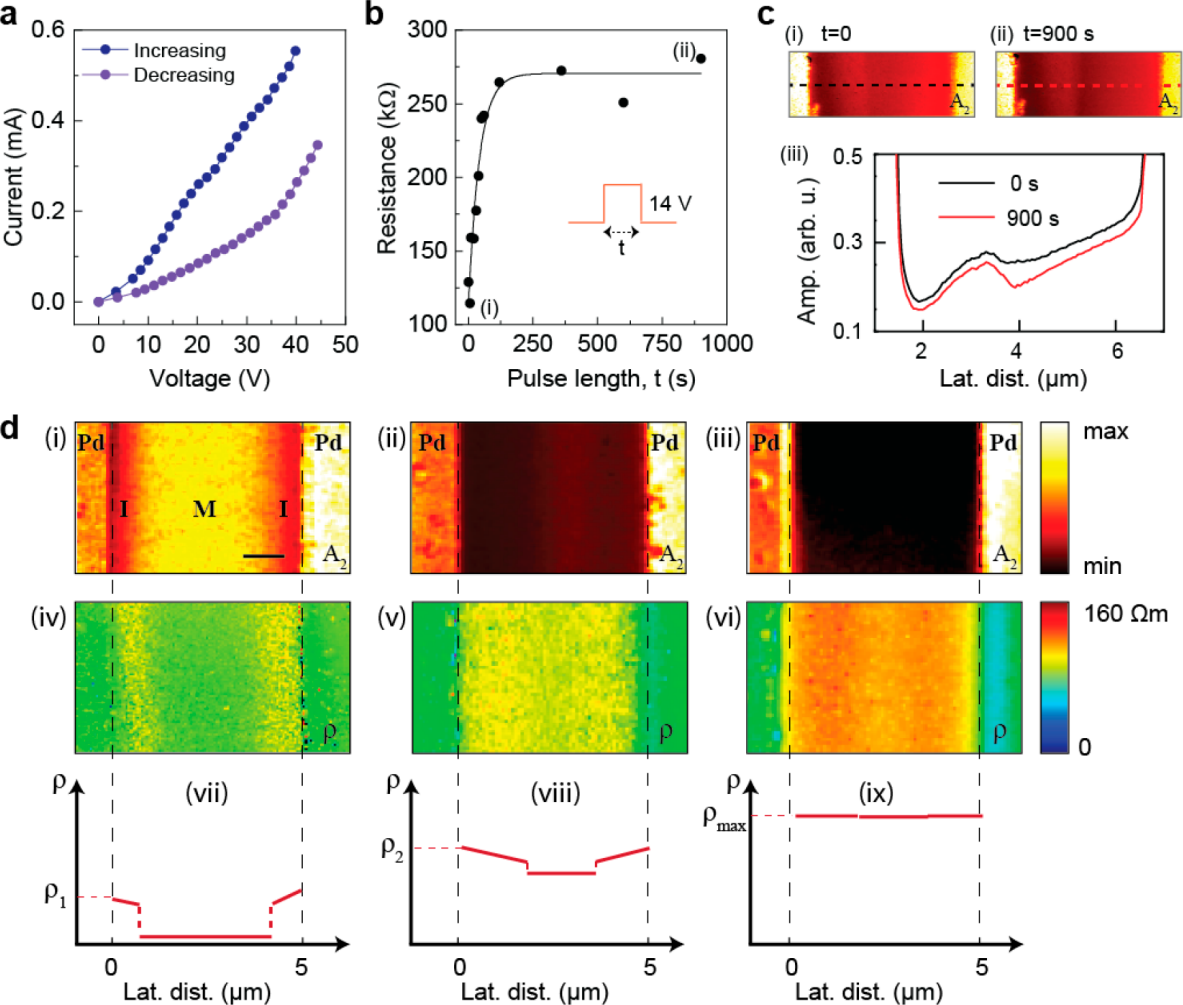
Effect of doping on global and local conductivity properties of H-NNO. (a) Current vs voltage relationship of H-NNO device with increasing (blue dots) and decreasing (purple dots) voltage across the device. (b) Change in global resistance across the device with voltage pulse duration (black dots); black solid line is guide to the eye. (c) Amplitude images taken at *t* = 0 s (i) and *t* = 900 s (ii) also shown in panel b, and the line profiles extracted from those two images (iii) along the horizontal dashed lines marked in panels c(i) and c(ii). (d) Near-field 2nd harmonic amplitude images of (i) lightly doped (40 Ω), (ii) medium doped (125 kΩ), and (iii) heavily doped (430 kΩ) global resistance states of the device. All near-field images were obtained at laser illumination frequency of ω = 953 cm^–1^. The near-field amplitude images in parts i–iii were converted to resistivity maps, shown in parts iv–vi. For this conversion, the Im(ε) values were obtained based on the calculations discussed in Figure 5 for H-NNO with different dopant levels. The plots vii–ix are resistivity profile schematics that correspond to the images in parts iv–vi. Scale bar in panel d(i) is 1 μm.

The device’s global (between electrodes) forward and reverse current (*I*) and voltage (*V*) relationship for *V* ranging between 0 and 45 V shown in Figure 6a resembles the hysteresis pattern reported for similar synaptic devices.^20^ The dependence of the global resistance on the pulse length is shown in Figure 6b. With increasing pulse length, global resistance exponentially increases and saturates at a level of ∼275 kΩ after the pulse length increased beyond 200 s. We display near-field amplitude images of two pulse lengths in Figure 6c, (i) *t* = 0 s and (ii) *t* = 900 s, and the corresponding line profiles extracted along the horizontal dashed lines are shown in Figure 6c(iii). After electrical pulsing, the change in the resistance distribution across the gap is demonstrated by the changes in the line profiles at *t* = 0 and *t* = 900 s. The decrease in amplitude signal is captured by the line profiles for 900 s, which indicates an increase in resistance after the applied pulses. For experiments presented in Figure 6b, the voltage pulse magnitude was kept at 14 V.

IR near-field images can be converted to resistivity maps, providing nanoscale electronic properties of the H-NNO channel. To that end, we imaged the Pd–NNO–Pd device at three different resistance levels that were produced by hydrogenation and application of pulsed E-fields between the electrodes. Figure 6d shows three subplots (i–iii) which show near-field s-SNOM amplitude images of H-NNO at three different resistance states: 40 Ω, 125 kΩ, and 430 kΩ. The images were obtained by using a laser excitation of ω = 953 cm^–1^. The near-field images were then converted to resistivity maps shown in Figure 6d(iv–vi). This is achieved by calculating the optical conductivity, σ_(RE)_, of the sample at each pixel using the equation  where ε_(IM)_ is the imaginary part of the dielectric function of the sample.^32−34^ To quantitatively extract the value of ε_(IM)_ at each pixel of the near-field image, we relied on our simulation method described above which provides the dielectric function of doped NNO at different dopant levels (see Methods section for details). The resulting resistivity maps (Figure 6e(iv–vi)) show a similar contrast profile as the near-field images and provide qualitative conducting states of the sample at a pixel level.

## Conclusion

Nanoscale infrared imaging, *in situ* high-resolution X-ray diffraction, and spectroscopy measurements revealed simultaneous structural and local conductivity modulation in H-NdNiO_3_ incurred by hydrogen dopants that can be actively controlled by applying electric fields. First-principles calculations, dielectric function simulations, and real-space infrared hyperspectral nanoimaging unveiled a range of vibrational modes that result from hydrogen doping. The results provide insight into electronic phase transitions mediated by ions at a length scale otherwise inaccessible in *operando* devices and how different dopants affect their electronic properties. Since the majority of memristive devices rely on conductivity modulation through local redistribution of various ionic defects, the ability to image the distribution of conducting domains will be valuable to understand how the local distribution affects global channel resistance. In the future, such correlation between local carrier distribution and global channel resistance can be useful for building physical models for neuromorphic device operation.

## Methods

### Sample Preparation

NNO films were deposited on 400 μm thick lanthanum aluminate (LaAlO_3_) substrates by the physical vapor deposition (PVD) sputtering technique. The thickness of the NNO films investigated in this study was 60 nm. For electrical contacts, Pd–Pd or Pd–Au electrode pads (100 μm × 100 μm) of thickness 80 nm were deposited on NNO films using electron beam lithography. Fabricated NNO devices were hydrogenated in a cell in a forming gas flow (hydrogen/nitrogen 5%) at 120 °C for ∼20 min while measuring the resistance between two Pd electrodes using an ohmmeter, until the measured resistance reached the desired level.

### Nanoimaging and Nanospectroscopy

A commercial (Neaspec GmbH) scattering type scanning near-field microscope (s-SNOM) that is based on a tapping mode AFM was utilized to perform the near-field imaging of the H-NNO devices. A metal coated cantilevered AFM tip oscillating at a frequency of Ω ∼ 280 kHz with a tapping amplitude of ∼100 nm was used to obtain both topography and near-field amplitude images. Spatial resolution is only limited by the tip-apex radius independent of the wavelength from visible to terahertz spectral range.^31,35−37^ For this work, we have used for nanoimaging a monochromatic quantum cascade laser of spatial frequency (ω) = 953 cm^–1^ and for nanospectroscopy a broadband light source in the spectral range of 600–2100 cm^–1^. As we presented in the SI Figure S7, the highest amplitude contrast between H-doped and undoped NNO was observed at the illumination of 953 cm^–1^. Hence, to clearly observe the dynamics at the nanoscale even under smaller doping/field changes, we used the illumination of 953 cm^–1^ for single frequency nanoimaging. The incident beam is focused on the tip–sample interface by a parabolic mirror at an angle of 45° to the sample surface and backscattered light from the interface was detected and demodulated at higher harmonics of tip resonance frequency using phase modulation interferometry (for single frequency imaging) and an asymmetric Fourier transform Michelson interferometer (for nanospectroscopy).

### X-ray Nanodiffraction Imaging

The X-ray nanodiffraction measurements were performed at beamline 7ID-C of the Advanced Photon Source.^38^ The 11.5 keV X-ray beam is focused by a Fresnel zone plate to a vertical spot size of 400 nm and a horizontal spot size of 1.2 μm full width at half-maximum (fwhm). The device sample was mounted vertically for a horizontal diffraction geometry, so the direction of the applied electric field was parallel to the vertical direction, and the long dimension of the gap of the device was aligned along the horizontal direction. The sample was raster scanned with the X-rays along the in-sample-plane directions, and the diffraction patterns were collected at various points in real space on the sample. Due to the broad rocking curve of the H-NNO film (SI Figure S4), the H-NNO diffraction peak across a large range of reciprocal space along the out-of-plane reciprocal lattice can be monitored at an incident angle of 15.7°. The corresponding average lattice constant can be derived from the 2θ angle of the diffraction peaks. The centroid of the diffraction peak was measured by fitting the H-NNO X-ray diffraction peak on the detector along the 2θ direction. The DC bias was applied by a Keithley 2612 source meter to the electrodes. The device reaches a steady state in about 10 min, which was confirmed by monitoring the current across the electrodes.

### Density Functional Theory (DFT) Calculations

All the density functional theory calculations were performed using the Vienna ab initio Simulation (VASP)^39^ package with the Perdew–Burke–Ernzerhof^40^ generalized gradient approximation (GGA)^41^ exchange correlation functional. The rotationally invariant form of GGA + U from refs (42 and 43) with *U* = 4.6 eV and *J* = 0.6 were used to treat the strong Coulomb repulsion among the Ni 3d electrons for NNO, where *U* is the on-site Coulomb parameter. All calculations used 600 eV as the plane wave cutoff energy. The total energy electronic convergence criterion was set at 10^–6^ eV. The structures were optimized using a conjugate gradient approximation^44^ as implemented in VASP until all the atomic forces were <0.01 eV/Å. The spin-polarized calculations were performed at the gamma points using a k-point density of 1000, where the k-point density was determined by multiplying the number of atoms in the simulation cell (*n*_atoms_) by the number of k-points (*n*_k-points_).^45^ The symmetry was turned off in all calculations. The metallic phases of NNO were considered with ferromagnetic initializations whereas in case of insulation phase we have considered the anti-ferromagnetic settings with T type anti-ferromagnetic ordering.^46^ Mixing parameters AMIX = 0.1 and BMIX = 0.001 were used to expedite the electronic convergence. Phonon modes and frequencies of the optimized structures at different levels of H concentration for both the phases were calculated using finite differences approaches.

### Molecular Dynamics (MD) Calculations

We simulate the dielectric response of pristine NNO using a Langevin dynamics methodology described by Hancock et al.,^30^ which models condensed matter systems as networks of damped oscillators with additional couplings to an oscillating external E-field. Systems simulated consisted of 5 coupled 20 × 20 arrays of atoms, each with periodic boundary conditions, all fixed at constant temperature. Typical time steps for integration were 10 fs, and the total integration time was 100 ps. To obtain precise mean values and calculate statistical error bars, 50 independent simulations were performed for each set of conditions. We set the resonant frequency (ω_0_) of the Nd–O bond ω_0,Nd–O_ = 150 cm^–1^. Similarly, we set ω_0,Ni–O_ = 125 cm^–1^. Damping parameter *b*/*m*_e_, where *m*_e_ is the mass of the bond charges in the simulation, was set to . We found that such a modeling scheme would be appropriately fast and flexible to explore different interactions and their resultant effects on the computed dielectric function. To fully investigate the role of H^+^ dopants in the dielectric modulation of NNO, we required an appropriate model for the dopant–lattice interactions. As such, we placed dopants randomly throughout the lattice interstitially at some predetermined concentration *n* and assumed for simplicity that such particles are static throughout the duration of the simulation. These dopants interact with surrounding lattice ions via a Lennard-Jones potential given by
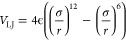
where ϵ describes the overall strength of the dopant interaction, σ similarly describes the spatial extent of the interaction, and *r* is the interparticle distance.

Different choices of the dopant interaction strength ϵ shift the associated peak position in frequency (Figure 3c), which bolsters this interpretation. Furthermore, an increase in the dopant concentration *n* increases the prominence of these dopant induced resonances shown in Figure 3d, as one would expect.

This computational procedure has allowed us to gain helpful insight into the manner in which dopants can impact the dielectric response of NNO in a way that is comparable to experiments. We then endeavored to increase the sophistication of our dopant modeling one step further by relaxing the static dopant restriction and adding in bond angle interactions between the dopants and the lattice. We, thus, introduce the three-body potential:
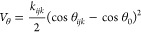
where *k*_*ijk*_ is the associated three-body interaction strength between a dopant *i* and particles *j* and *k*, θ_*ijk*_ is their instantaneous bond angle, and θ_0_ is their bond angle at dopant equilibrium. Therefore, we have a combined dopant interaction potential represented by
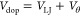
As explained above, we scan through different selections of *k*_*ijk*_ to show how the introduction of a bond-angle interaction might change the resonance landscape seen in the dielectric function. In Figure 3e, we have represented two spectra: one when *k*_*ijk*_ = 0, and one where *k*_*ijk*_ ≠ 0. In both cases, we kept ϵ the same to better illuminate the role of the bond-angle interactions on producing a more complex dielectric response as seen in experiments. We see the emergence of three dopant peaks when *k*_*ijk*_ ≠ 0 and only one otherwise. The addition of these three-body interactions allows for more complex interactions in the system, which is represented by the more numerous peaks in the spectra. This indicates that these interactions drive much of the unique and surprising features seen in the optical response of H^+^-doped NNO.

## Data Availability

Data will be made available upon reasonable request to the corresponding author.

## Supporting Information Available

Expansion of H-NNO film with reference to the electrode under the influence of applied electric field, measured changes in height and amplitude with respect to scanning position induced by a finite electric field, nanoscale variation of near-field amplitude of H-NNO, HRXRD rocking curves of the Pd-Pd device inside the gap, Calculated near-field amplitude and phase spectra of NNO with different hydrogen doping levels, phonon density of states for pristine and H_0.25_-NNO, near-field amplitude images of H-NNO with a Pd electrode at different laser illumination frequencies, and real-space nanoscale imaging of hydrogen migration in topography and s-SNOM amplitude images of doped H-NNO obtained with illumination ν = 953 cm^–1^ as a function of decreasing applied E-field (PDF)
