# Supplementary material for: Infrared Nanoimaging of Hydrogenated Perovskite Nickelate Memristive Devices
Source: ACS Nano. 2024 Jan 10;18(3):2105–16. doi: 10.1021/acsnano.3c09281 (PMC10811663; doi:10.1021/acsnano.3c09281)
Supplement: Supplementary file 1 — nn3c09281_si_001.pdf [file nn3c09281_si_001.pdf]

Supporting Information

Infrared Nanoimaging of Hydrogenated Perovskite Nickelate Memristive Devices

Sampath Gamage[1], Sukriti Manna[2,3], Marc Zajac[4], Steven Hancock[5], Qi Wang[6], Sarabpreet Singh[1], Mahdi Ghafariasl[1], Kun Yao[7], Tom E. Tiwald[8], Tae Joon Park[6], David P. Landau[5], Haidan Wen[4,9], Subramanian K.R.S. Sankaranarayanan[2,3], Pierre Darancet[2,10], Shriram Ramanathan[6,11] and Yohannes Abate[1*]

[1]Department of Physics and Astronomy, University of Georgia, Athens GA 30602, USA

[2]Center for Nanoscale Materials, Argonne National Laboratory, Lemont, IL 60439, USA

[3]Department of Mechanical and Industrial Engineering, University of Illinois, Chicago, IL, 60607, USA

[4]Advanced Photon Source, Argonne National Laboratory, Lemont, Illinois 60439, USA

[5]Center for Simulational Physics and Department of Physics and Astronomy, University of Georgia, Athens GA 30602, USA

[6]School of Materials Engineering, Purdue University, West Lafayette, IN 47907, USA

[7]School of Electrical and Computer Engineering, University of Georgia, Athens GA 30602, USA

[8]J.A. Woollam Co., Inc., Lincoln, NE 68508, USA

[9]Materials Science Division, Argonne National Laboratory, Lemont, IL 60439, USA

[10]Northwestern Argonne Institute of Science and Engineering, Evanston, IL 60208, USA

[11]Department of Electrical & Computer Engineering Rutgers, The State University of New Jersey, Piscataway, NJ 08854, USA

*Corresponding author E-mail: yohannes.abate@uga.edu

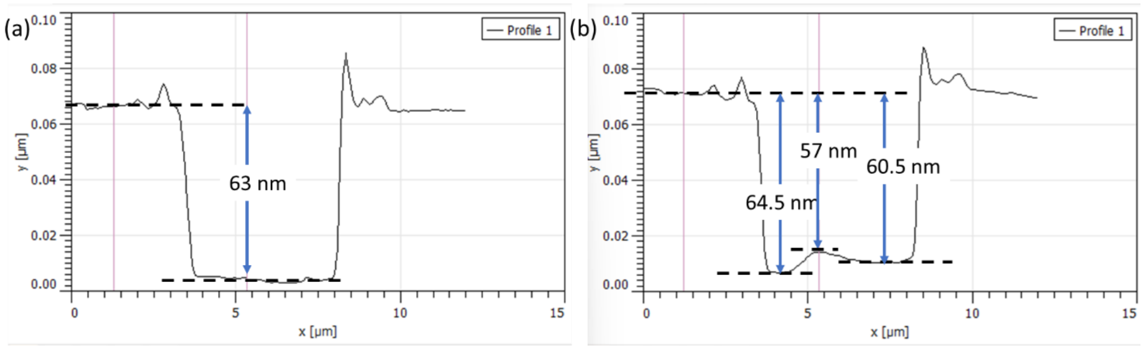

Figure S1. Expansion of H-NNO film with reference to the electrode under the influence of applied electric field. (a) Relative height from the H-NNO film to the top of the electrode without any electric field applied. (b) Relative height from the different regions of the H-NNO film when electric field of 1.46 Vμm^{-1}is applied from left to right across electrodes.

Increase in thickness (%) of H-NNO film under the E-field was calculated as follows:

Max. change in height	= (63-57) nm
	= 6 nm
Grown thickness of NNO film	≈ 100 nm
% Increase in thickness	≈ 6%

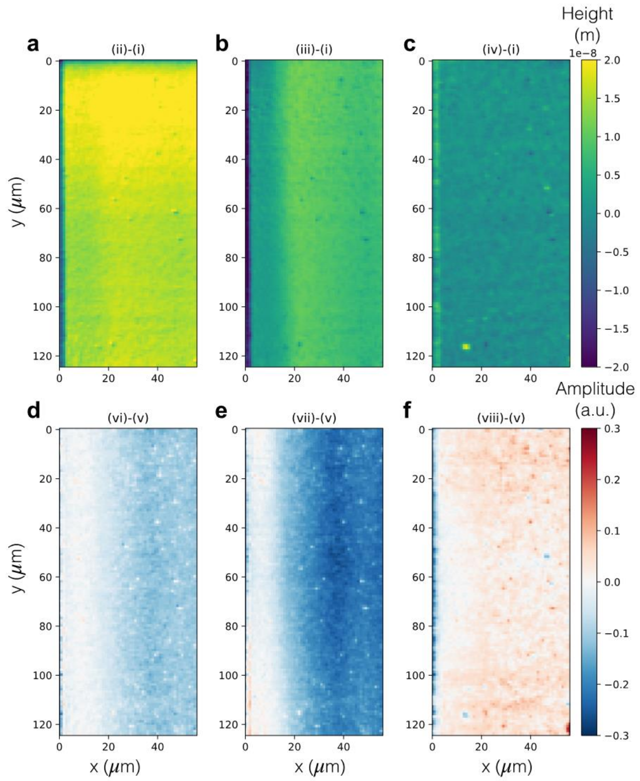

Figure S2. Measured changes in height (top) and amplitude (bottom) with respect to scanning position induced by a finite electric field. Top: Difference in the height profiles measured through topography between E=1.12 Vμm^{-1} (a), 1.46 Vμm^{-1} (b) and final, 0 Vμm^{-1} (c) and the initial, no field configuration. Bottom (d-e): Same for s-SNOM images. Calculated Pearson correlation are 0.44 (between a and d), 0.2 (between b and e), and 0.56 (between c and f).

The Pearson correlation coefficient was calculated between profile differences extracted from raw atomic force microscopy (AFM) and scanning near-field optical microscopy (SNOM) image data. AFM provides surface height profiles under varying electric field strengths, and SNOM gives optical amplitude profiles for the same field conditions. The procedure included generating height/amplitude difference profiles by subtracting a "no field" reference profile from each raw profile obtained under an applied field. The differences, one from AFM and one from SNOM, for each field strength were then subjected to a Pearson correlation algorithm. This algorithm involved centering the datasets by subtracting the mean, calculating covariance, and normalizing by their standard deviations. The resulting Pearson coefficient quantified the correlation between surface morphology differences measured by AFM and optical amplitude shifts measured by SNOM. The approach focused on deriving and correlating differences from raw profile data.

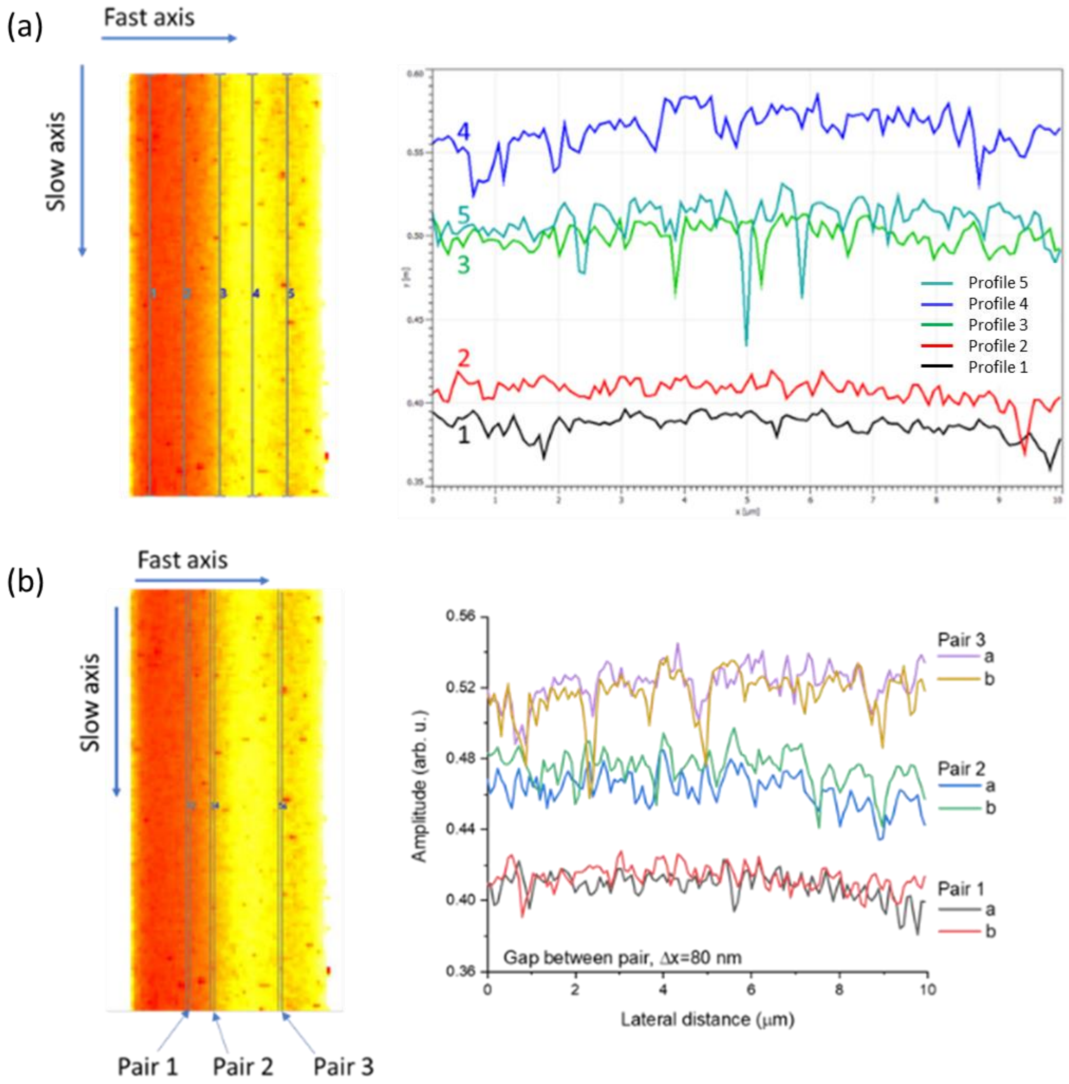

Figure S3. Nanoscale variation of near-field amplitude of H-NNO.

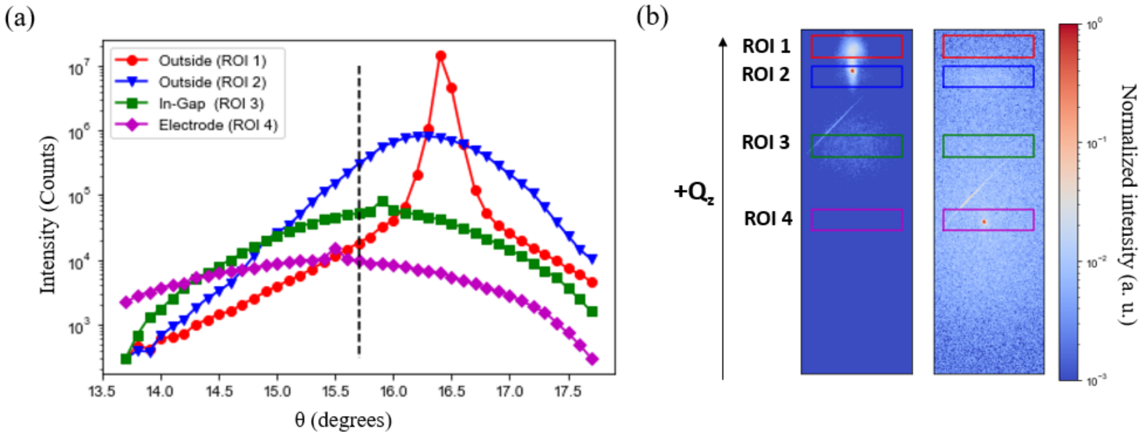

Figure S4. HRXRD rocking curves of the Pd-Pd device inside the gap, with a black dashed line indicating the sample angle ($\Theta = 15.7^{\circ}$) at which Figure 2 was measured. Rocking curves were taken in 3 different spatial locations: outside the device, in the device gap, and under the device electrode. The intensity at 4 different regions of interest (ROIs) on the detector were summed and plotted as a function of sample angle. (b) Representative x-ray diffraction images taken from the in-gap rocking curves at 16.3° (left) and from rocking curves taken underneath the electrode at 15.5° (right) with the various region of interests (ROIs) that were used in Figure S3a shown as different colored rectangles on the diffraction images. The linear, diagonal feature on the diffraction pattern is diffuse scattering from the substrate.

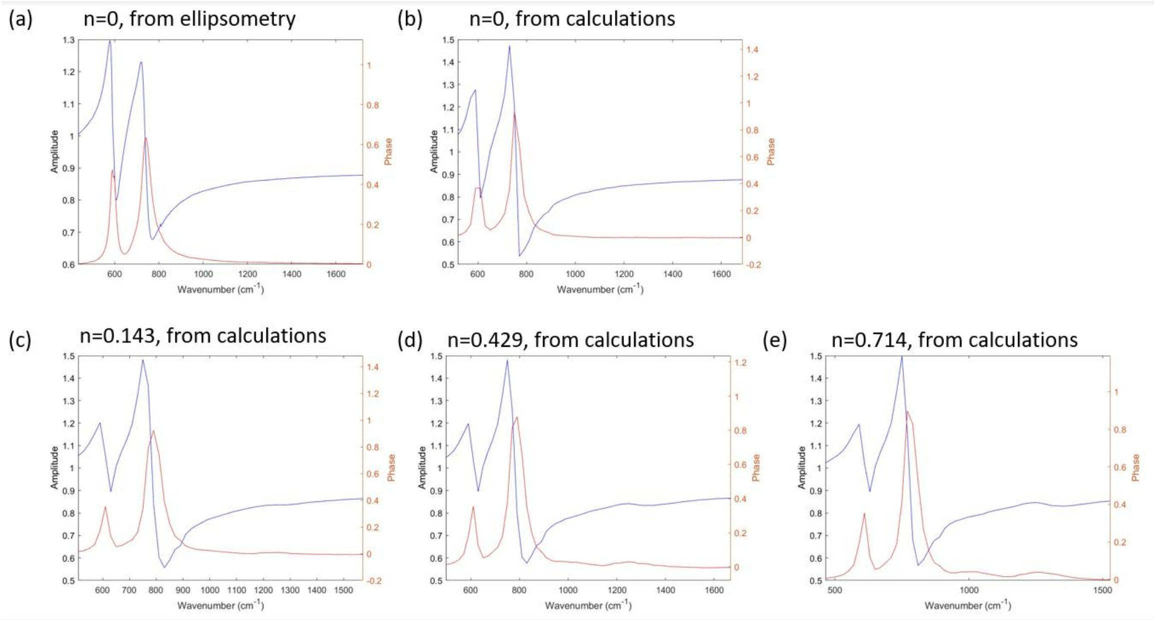

Figure S5. Calculated near-field amplitude and phase spectra of NNO with different hydrogen doping levels (*n*), using dipole model.

The near-field amplitude and phase of NNO samples with different doping levels (*n*) were calculated using extended finite dipole model and presented in Figure S5. First, we used the dielectric function values of undoped NNO obtained from ellipsometry to calculate the amplitude and phase and resulted spectra are shown in Fig. S5a. Then we used the dielectric values obtained from molecular dynamics calculations to generate the amplitude and phase spectra for NNO with doping levels, n=0 (b), 0.143 (c), 0.429 (d) and (e) 0.714.

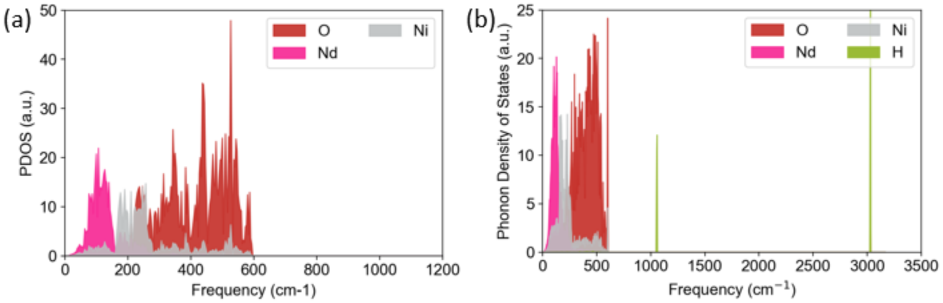

Figure S6. (a) Phonon density of states for (a) pristine and (b) $H_{0.25}$-NNO.

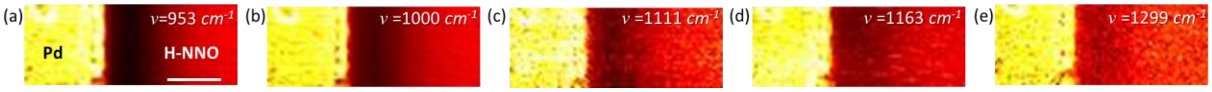

Figure S7. The near-field amplitude images of H-NNO with a Pd electrode at different laser illumination frequencies: 953, 1000, 1111, 1163 and 1299 cm^{-1}, respectively, from (a) to (e). Scale bar in (a) is 1 μm. The highest amplitude contrast was observed at the illumination of 953 cm^{-1}. Hence, to clearly observe the dynamics at nanoscale even under smaller doping/field changes, we imaged at 953 cm^{-1}.

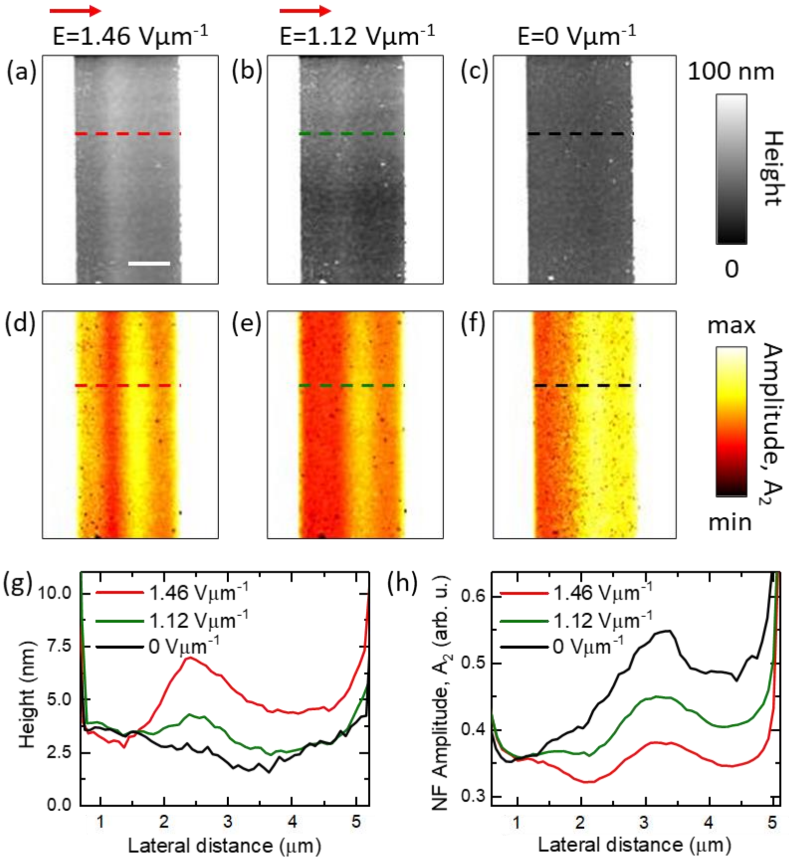

Figure S8. Real-space nanoscale imaging of hydrogen migration in topography (a-c) and s-SNOM amplitude (d-f) images of doped H-NNO obtained with illumination ν= 953 cm^{-1} as a function of decreasing applied E-field. Topography (a-c) and applied E-field dependent near-

field amplitude images (d-f) for E= 1.46, 1.12 and 0 Vμm^{-1}, respectively. Scale bar in (a) is 2 μm. Line profiles of topography (g) and amplitude (h) along the lines marked on the topography and amplitude images shown in (a-f).
